# Valorization of Tomato Processing by-Products: Fatty Acid Extraction and Production of Bio-Based Materials

**DOI:** 10.3390/ma11112211

**Published:** 2018-11-07

**Authors:** José J. Benítez, Paula M. Castillo, José C. del Río, Manuel León-Camacho, Eva Domínguez, Antonio Heredia, Susana Guzmán-Puyol, Athanassia Athanassiou, José A. Heredia-Guerrero

**Affiliations:** 1 Instituto de Ciencia de Materiales de Sevilla, Centro Mixto CSIC-Universidad de Sevilla, Américo Vespucio 49, E-41092 Seville, Spain; pmcasher@gmail.com; 2 Instituto de Recursos Naturales y Agrobiología de Sevilla-CSIC, Avenida Reina Mercedes 10, 41012 Seville, Spain; delrio@irnase.csic.es; 3 Instituto de la Grasa, CSIC, 41006 Seville, Spain; mleon@ig.csic.es; 4 Instituto de Hortofruticultura Subtropicaly Mediterránea La Mayora, Universidad de Málaga-CSIC, E-29071 Málaga, Spain; edominguez@eelm.csic.es (E.D.); heredia@uma.es (A.H.); 5 Departamento de Biología Molecular y Bioquímica, Universidad de Málaga, E-29071 Málaga, Spain; 6 Smart Materials, Istituto Italiano di Tecnologia, 16163 Genova, Italy; susana.guzman@iit.it (S.G.-P.); athanassia.athanassiou@iit.it (A.A.)

**Keywords:** agricultural by-product, tomato pomace, valorization, fatty acids, bio-based polymers

## Abstract

A method consisting of the alkaline hydrolysis of tomato pomace by-products has been optimized to obtain a mixture of unsaturated and polyhydroxylated fatty acids as well as a non-hydrolysable secondary residue. Reaction rates and the activation energy of the hydrolysis were calculated to reduce costs associated with chemicals and energy consumption. Lipid and non-hydrolysable fractions were chemically (infrared (IR) spectroscopy, gas chromatography/mass spectrometry (GC-MS)) and thermally (differential scanning calorimetry (DSC), thermogravimetric analysis (TGA)) characterized. In addition, the fatty acid mixture was used to produce cutin-based polyesters. Freestanding films were prepared by non-catalyzed melt-polycondensation and characterized by Attenuated Total Reflected-Fourier Transform Infrared (ATR-FTIR) spectroscopy, solid-state nuclear magnetic resonance (NMR), DSC, TGA, Water Contact Angles (WCA), and tensile tests. These bio-based polymers were hydrophobic, insoluble, infusible, and thermally stable, their physical properties being tunable by controlling the presence of unsaturated fatty acids and oxygen in the reaction. The participation of an oxidative crosslinking side reaction is proposed to be responsible for such modifications.

## 1. Introduction

Driven by the worldwide demand for primary foodstuff supply, crop production has been pushed to very high rates, while the consumption of processed food products increases every year. The residues generated by such activities reach ~1.3 billion tons at the global level (~1/3 of the food produced worldwide), of which ~88 Mt are estimated to be produced in Europe [1,2]. These residues have been traditionally wasted in landfills and bodies of water, causing important environmental and health issues. In addition, the demands of water, land-use, energy, chemicals and materials, as well as the emissions of greenhouse gases during production of food that finishes wasted, increase considerably the environmental impacts of such residues [3]. Currently, this underutilized, inexpensive biomass is regarded as an accessible and worthwhile feedstock for the fabrication of valuable products such as edibles for humans and livestock, bio-fertilizers, biofuels, and manufactured commodities [4].

Tomato fruit is a good example. According to the Food and Agriculture Organization Corporate Statistical Database (FAOSTAT), it is the second largest primary vegetable crop with a world production of 177 million tons in 2016 [5], 24% of which is processed to obtain paste, purée, sauce or juice, with this percentage being increased by up to 60% in developed areas. Tomato pomace is the resulting residue and consists of peels, seeds, and fibers. On a dry basis, it represents about 2–3% of the fresh processed weight [6,7,8] and constitutes a potential global renewable feedstock ranging from 0.8 to 1.3 Mtons/year. Tomato pomace can be used as a supplement for animal feeding, and, due to its high protein content (~10–20% *w*/*w*), has also been considered for human consumption [9]. In addition, other applications related to the production of energy and tin corrosion inhibition have been proposed for this agricultural by-product [10,11]. Nowadays, it is partially exploited to obtain lycopene, a powerful natural antioxidant used in food, pharmaceutical, and cosmetic products [12], although it is mainly burnt or wasted, which implies additional labor costs for removal and disposal.

Plant cutin is the main component (up to 80% *w*/*w*) of the skin fraction of tomato pomace. It is a non-toxic, biodegradable, waterproof, UV-blocking, amorphous, insoluble, and infusible biopolyester made of esterified C_16_ and C_18_ hydroxyacids [13]. Such properties have revealed the potential of cutin-based polymers and, in fact, cutin is considered a realistic raw material to produce alternative materials to conventional plastics for specific uses, as reported by the United Nations [14]. Recently, its valorization for the production of bioplastics has been reviewed [15], applications in food packaging being the most highlighted but also with potential for biomedical use [16,17,18]. The insolubility and thermosetting features of such cross-linked polyhydroxylated aliphatic esters have been exploited to develop inert and heat-resistant coatings and composites [19,20,21,22]. Thus, internal cutin-based coatings for food cans have been proposed as a non-toxic and sustainable alternative to bisphenol A (BPA) resins [16] and the processing of the skin fraction of the tomato pomace for this purpose has been previously addressed [23]. However, no detailed description and characterization of the polymeric material obtained from such monomeric mixture was reported. Furthermore, no analogous studies regarding the processing of the integral tomato pomace (i.e., skin, seeds, and fibrous rests) are currently available.

This article aims to optimize the tomato pomace fractionation to control the fatty acid extraction. These fatty acids are used to produce bio-based polyesters. To preserve the innocuousness of the polymer formed and increase the sustainability of the overall process, we have used an organic solvent-free non-catalyzed melt-polycondensation reaction. To reduce costs and gain attractiveness for a large-scale production, the polymerization is performed directly in air. The influence of the oxidative atmosphere is evaluated and discussed by comparison with samples obtained in an inert (N_2_) environment.

## 2. Materials and Methods

### 2.1. Materials

Tomato pomace was provided by Conservas Martinete S.A. (Puebla de la Calzada, Badajoz, Spain). Chemicals used: sodium hydroxide, hexane, methanol, and hydrochloric acid were analytical grade and purchased from Sigma-Aldrich (Milan, Italy). Water was Milli-Q (Millipore, Milan, Italy) grade.

### 2.2. Tomato Pomace Conditioning

Highly wet by-products were in situ sun dried for 2–3 days and stored in hermetic plastic bags until further drying (4 h later) inside a forced air oven at 60 °C for 48 h. The dry residue was partially crushed and kept in a freezer at −4 °C until used. The crushed residue was first treated with a refluxing mixture of hexane:methanol (3:1, *v*/*v*) under vigorous stirring to remove waxes and facilitate the subsequent alkaline hydrolysis (the use of hexane is currently under restriction in the food processing industry and alternative procedures are currently under consideration). The washed solid was dried overnight in an oven at 60 °C, cooled, and ground with a rotor mill equipped with a stainless steel sieve ring with 0.08 mm trapezoidal perforations.

### 2.3. Hydrolysis and Recovery of Fractions

**Figure 1:**
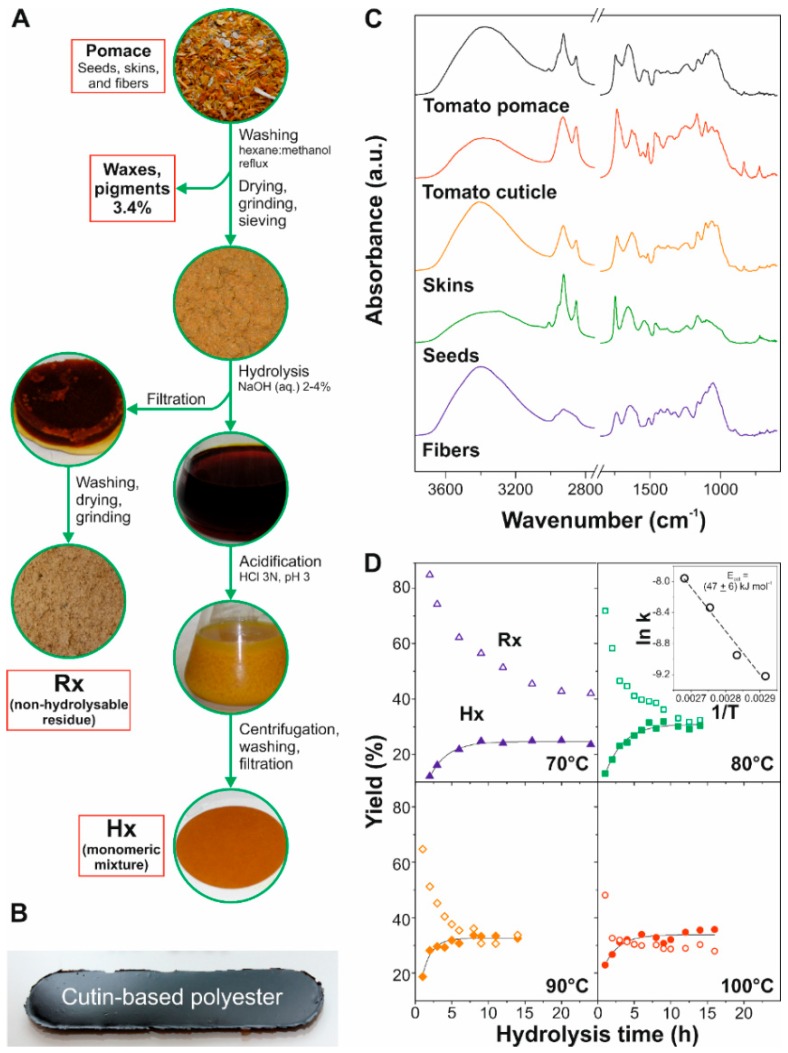
(**A**) Tomato pomace conditioning and processing to isolate the non-hydrolysable fraction (Rx) and the monomeric mixture (Hx). (**B**) Picture of a typical cutin-based film. (**C**) Infrared spectra of fibers, seeds, skins, tomato cuticle, and tomato pomace. (**D**) Recovery of the non-hydrolysable (Rx, empty symbols) and monomer mixture (Hx, filled symbols) in the alkaline hydrolysis of tomato pomace as a function of temperature and reaction time. The inset shows the ln k versus 1/T plot that allows the calculation of the activation energy.

Dewaxed ground tomato pomaces (~3 g) were treated with aqueous NaOH solutions (150 mL) at concentrations from 0.5 to 1.0 M in a round bottom flask with continuous stirring and equipped with a Dimroth condenser. Hydrolysis time ranged from 1 to 24 h and temperatures were 70, 80, 90, and 100 °C. At the lowest NaOH concentration used, the base was calculated to be in an excess of ~15 times. The non-hydrolysable residue (Rx) obtained after the alkaline treatment was separated by filtration and quantified gravimetrically after exhaustive washing with Milli-Q water and drying at 80 °C for 24 h. The monomeric mixture (Hx) was recovered from the hydrolysis filtrate by acidification with a 3 N HCl aqueous solution until pH 3. The precipitate was separated by centrifugation and re-dispersed in water for three times. The final product was isolated by filtration and vacuum dried for 3 days. The absence of NaCl residues was checked by X-ray diffraction. Figure 1A shows a graphical scheme of these steps.

### 2.4. Chemical Characterization of Tomato Pomace and its Components and Fractions

#### 2.4.1. Infrared Spectroscopy

Samples were characterized by infrared spectroscopy with a FTIR spectrometer (FT/IR-6200, JASCO, Madrid, Spain) in transmission mode. Pellets containing 1.5 mg of sample dispersed in 150 mg of dry KBr were prepared and 32 scans accumulated using a deuterated triglycine sulfate (DTGS) detector operating at 4 cm^−1^ resolution.

#### 2.4.2. Fatty Acid Composition

The analytical composition of the Hx fractions (from either pomace or isolated seeds) was determined by gas chromatography/mass spectrometry (GC-MS) analysis. Around 2 mg of each sample were dispersed in 2 mL chloroform: methanol (3:1, *v*/*v*), sonicated for 10 min, and then passed through syringe filters to obtain a clear solution. The extracts were evaporated to dryness under a N_2_ stream and subsequently silylated with 0.25 mL *N*,*O*-bis-(trimethylsilyl)trifluoroacetamide (BSTFA, Supelco) in the presence of 0.05 mL pyridine (at 70 °C for 2 h) before GC-MS analyses. The analyses were performed with a Shimadzu GC-MS QP2010 Ultra, using a fused-silica DB-5HT capillary column (30 m × 0.25 mm i.d., 0.1 μm film thickness) from J&W Scientific. The oven temperature was kept at 50 °C for 1.5 min and then raised to 90 °C at 30 °C/min and maintained for 2 min. Next, it was raised to 250 °C at 8 °C/min followed by an isothermal period of 15 min at the latter temperature. The injection was performed at 250 °C and the transfer line was kept at 300 °C. Helium (1 mL/min) was used as the carrier gas. Compounds were identified by comparing their mass spectra and retention times with those of authentic standards. For this, a series of standard fatty acids (from Sigma-Aldrich, Steinheim, Germany) was used including: (i) saturated fatty acids such as tetradecanoic, hexadecanoic, and octadecanoic acids; (ii) unsaturated fatty acids such oleic (*cis*-9-octadecenoic) and linoleic (*cis*,*cis*-9,12-octadecadienoic) acids; (iii) dicarboxylic acids such as hexadecanedioic and octadecanedioic acids; (iv) hydroxyfatty acids such as 16-hydroxyhexadecanoic. The rest of the fatty acids were identified by comparison of their mass spectra with those present in the Wiley and National Institute of Standards and Technology (NIST) mass spectral database and with those reported in the literature, and by analysis of their mass fragmentation patterns. Quantification was obtained from the total-ion peak areas. The summed areas were normalized and expressed as molecular percentages.

### 2.5. Polymer Synthesis

Polymer films from Hx fractions were prepared by melt-polycondensation without catalyst using a sealed carbon doped Teflon mold. The reaction was conducted at 150 °C under dry air or N_2_ flow for periods ranging from 3 to 20 h. Dark brown uniform films ~350 µm thick were detached from the mold, Figure 1B.

### 2.6. Polymer Characterization

#### 2.6.1. Chemical Characterization

Infrared spectra were collected with a FT-IR spectrometer (FT/IR-6200, JASCO, Madrid, Spain) in Attenuated Total Reflected (ATR) mode. A single reflection accessory (MIRacle ATR, PIKE Technologies, Madrid, Spain) coupled to a liquid N_2_ cooled mercury cadmium telluride (MCT) detector was used. Spectra were recorded in the 4000 to 600 cm^−1^ range at 4 cm^−1^ resolution after the accumulation of 50 scans.

Solid-state ^1^H and ^13^C magic angle spinning (MAS)-NMR proton decoupling single-pulse spectra were obtained with a Bruker Avance DRX-400 spectrometer (Bruker, Madrid, Spain) using a magnetic field of 9.36 T and equipped with a multinuclear probe. Minced samples were packed in 4 mm Ø zirconia rotors and spun at 10 KHz. The spectra were acquired at a frequency of 100.61 MHz, using a π/6 pulse width of 2.5 μs and a pulse space of 10 s to ensure full relaxation. Chemical shifts are referenced to tetramethylsilane.

#### 2.6.2. Thermal Characterization

DSC thermograms were acquired with a DSC Q20 (TA Instruments, Guyancourt, France) from −70 to 150 °C under dry N_2_ flow (50 mL/min) at 10 °C/min, using non-hermetic aluminum pans. For polymers, accurately weighed small pieces (about 4 mg) were placed in the pans and cooled to −70 °C. After that, a heating-cooling-heating cycle was performed. The glass transition temperature (*T*_g_) was calculated from the second heating using the inflection method.

TGA profiles of polymers were obtained with a SDT Q600 TGA/DSC analyzer (TA Instruments, Guyancourt, France). Samples (~6 mg) were heated from room temperature (RT) to 500 °C at 5 °C/min under N_2_ flow (100 mL/min).

#### 2.6.3. Mechanical Characterization

Tensile tests of polymers were performed with a MTS Criterion 42 machine equipped with a 10 N load cell. Rectangular pieces of 7 mm × 20 mm and about 350 μm thick were brought to rupture at a constant deformation rate of 5 mm/min at room conditions. Stress values were calculated using the specimen cross-section under no applied load and the Young’s modulus from the initial slope of the stress-strain curves. The parameter values presented are the average of a minimum of 5 measurements.

#### 2.6.4. Surface Hydrophobicity

Static Water Contact Angles (WCA) of the polymeric films were determined with an Attension TL100 Optical Tensiometer (Attension, Helsinki, Finland) by the sessile drop method using a 3 μL Milli-Q grade water drop. At least five locations per sample were assayed and the values presented correspond to their average. Prior to measurements, specimens were rinsed with isopropanol and dried overnight.

## 3. Results and Discussion

### 3.1. Characterization of Tomato Pomace Fractions

The fraction content of dry tomato pomace was calculated by separating its components (see Table 1). Seeds were the main fraction with a 55% (*w*/*w*) followed by peels (30%, *w*/*w*) and fibers (15%, *w*/*w*). This fractionation is comparable to others [9,24] though Al-Wandawi et al. reported a slightly higher skin content (~41%). Differences may be due to fruit variability and fractioning process. According to such literature results and others [25,26,27], the composition of each fraction is listed in Table 1 and from them, the content in the dry tomato pomace has been calculated. Thus, considering tomato pomace as a whole, main components were fatty acids (34%), polysaccharides (34%), and proteins (16%).

The different fractions of tomato pomace were chemically characterized by infrared spectroscopy and compared with isolated ripe tomato cuticles (see Figure 1C and Table S1). The infrared spectrum of fibers was typical of amorphous polysaccharides with characteristic bands at 1052 and 1079 cm^−1^ (C–OH and C–C stretching modes), 1104 cm^−1^ (ring vibration), 1161 cm^−1^ (ν_a_(C–O–C) glycosidic bond), and 896 cm^−1^ (C_1_, anomeric vibrations) [28]. Bands at 989 and 896 cm^−1^ were assigned to cellulose, while the position of the main absorption at 1052 cm^−1^ was representative of hemicellulose. In particular, the shoulder at 2965 cm^−1^ and the peak at 1377 cm^−1^ were characteristic of rhamnose blocks [29]. Additionally, bands at 1247, 1509, and 1597 cm^−1^ were attributed to aromatic units of lignin [30]. A peak at 1737 cm^−1^ was observed and associated with C=O stretching mode of esters. Since the aliphatic contribution was very low, as revealed by the low intensity of the ν(CH_2_) bands, the participation of cutin and triglycerides was considered negligible. However, the contribution of aromatic esters from the attachment of phenolic compounds to polysaccharides cannot be excluded, as proposed elsewhere [31]. On the other hand, the infrared spectrum of tomato seeds showed several peaks corresponding to a ligno-cellulosic fraction (1055, 1092, 1161, and 1242 cm^−1^) and proteins (1655, 1541, and 1237 cm^−1^, assigned to amide I, II, and III, respectively) [32]. There was also an important esterified lipid content (i.e., triglycerides), as revealed by intense ν(CH) peaks at 2855 and 2925 cm^−1^ as well as ν(C=O) at 1746 cm^−1^. The band at 3009 (ν(C=C–H)) and, to a lesser extent, the shoulder around 1620 cm^−1^ (ν(C=C)) also indicated the presence of unsaturated fatty acids. These results are in good agreement with reported tomato seed composition, which contains comparable amounts of crude fiber, proteins, and fats (~80% unsaturated fatty acids) [9,24,26,33]. Finally, skins displayed characteristic bands of biopolyester cutin (ν(C=O) at 1733 cm^−1^ and asymmetrical and symmetrical ν(C–O–C) at 1165 and 1103 cm^−1^, respectively) and a polysaccharide fraction (coupled backbone vibrations between 900–1150 cm^−1^) [32,34]. The profile of this latter region slightly differed from those of seed and fiber, indicating a different polysaccharide distribution. Thus, the coupled ν(C–O) and ν(C–C) band at 1029 cm^−1^ may result from the higher presence of pectin in the skin fraction of pomace [35]. Indeed, the relative amount of pectin is comparable to cellulose and hemicellulose in the cuticle of ripe tomato fruit cuticles [27]. When compared to enzymatically isolated tomato cuticles, the higher intensity of polysaccharide bands (1035, 1065, and 1460 cm^−1^) can be due to the presence of additional cell walls in the skin fraction. The presence of phenolic compounds (e.g., *p*-coumaric acid) and flavonoids was revealed by absorptions at 1628, 1608, 1550, and 1441 cm^−1^ [34].

### 3.2. Kinetics and Activation Energy of the Alkaline Hydrolysis

The alkaline hydrolysis of the dewaxed and ground tomato pomace was studied at several temperatures and reaction times, Figure 1D. The yield percentages of the non-hydrolysable residue (Rx) and the soluble extract (Hx) depended on both hydrolysis time and temperature in an opposite way. For instance, after 24 h of hydrolysis, the values decreased from 41 to 32% for Rx at 70 and 100 °C, respectively. By contrast, Hx was increased from 25 to 32% at 70 and 100 °C, respectively. With these data, and considering an excess of base during the hydrolysis, the extract recovery (Hx) was fitted to a pseudo first-order kinetic Equation (1):

Hx = Hx^max^ (1 − e^−k′t^) (1)

where (Hx^max^) is the recovery at infinite time and (k′) the apparent rate constant that includes the rate constant (k) and the NaOH concentration (k′= k [NaOH]). From this analysis, the predicted maximum recovery (Hx^max^) was calculated to be ~31% (*w*/*w*) of the dry tomato pomace, close to the 34% predicted from the recuperation fatty acids from seeds and cutin, Table 1. On the other hand, as expected, the rate constants were increased with the temperature. Their values (k) were 1.0, 1.3, 2.4, and 3.5 × 10^4^ L mol^−1^ s^−1^ for 70, 80, 90, and 100 °C, respectively.

The Arrhenius plot of the ln k vs 1/T resulted in an activation energy of (47 ± 6) kJ mol^−1^ for the alkaline treatment, inset of Figure 1D. This energy is similar to those reported for the hydrolysis of ethyl acetate in water (45.2 ± 0.7 kJ mol^−1^) [36] and lower than the depolymerization of solid poly(ethylene terephthalate) in alkaline solution (66–69 kJ mol^−1^) [37].

Considering the recovery data, and to reduce costs associated with chemicals and energy consumption, routine hydrolysis conditions were set to 100 °C for 6 h using NaOH 0.5 N.

### 3.3. Chemical Analysis of Rx and Hx

The non-hydrolysable solid (Rx) recovered as the supernatant after the complete alkaline hydrolysis represented about 31–32% of the dry pomace. This amount is consistent with the polysaccharide content of the fractions of tomato pomace (34%) estimated from Table 1. Such non-hydrolysable Rx residue was analyzed according to standard protocols [38] and contained 7.5% lignin, 17% hemicellulose and 70% cellulose (5.5% ash). The low content of lignin makes this by-product very suitable for mild hydrolytic treatments and the production of bioalcohols by fermentation, which becomes and additional aspect to consider in the complete valorization of tomato pomace.

Hx from pomace was characterized by infrared spectroscopy and compared to Hx from seeds and skin, that is, the analogous fractions obtained by the treatment of isolated seeds and skins in the same conditions, Figure S1. Results indicated that Hx pomace was constituted by unsaturated (from seeds) and polyhydroxylated (from skins) fatty acids. To quantify these results and determine the accurate chemical composition of the fatty acids, Hx pomace and Hx seed fractions were analyzed by GC-MS, Table 2. As observed, Hx seed was dominated by unsaturated acids, in particular linoleic acid (~53%) followed by oleic acid (~28%). These percentages are in good agreement with those described elsewhere [24,33,39]. In Hx pomace, dihydroxylated C_16_ acids predominate (mainly 10,16-dihydroxypalmitic, ~43%) as expected from reported data for cutin in tomato fruit [40].

### 3.4. Thermal Characterization of Pomace Components and Hx Fractions

**Figure 2:**
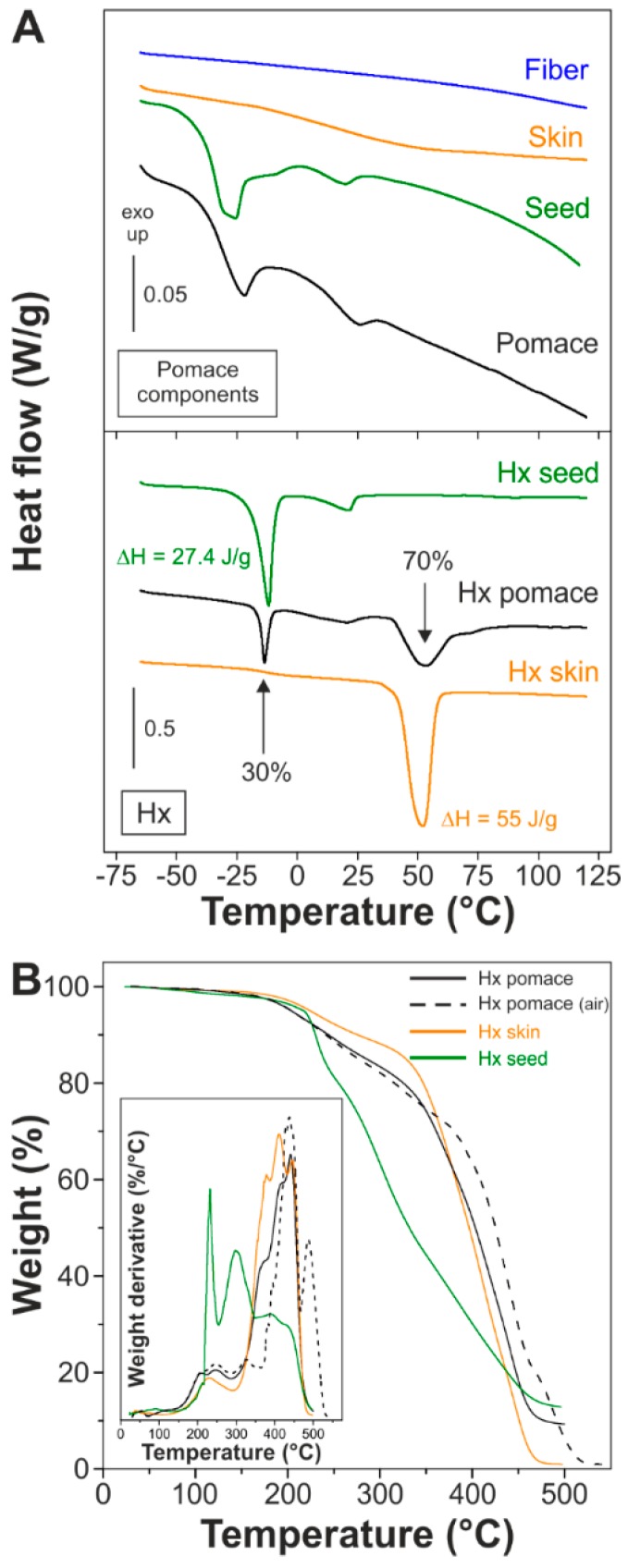
(**A**) DSC of isolate tomato pomace constituents and their hydrolysable fractions (Hx). (**B**) TGA of Hx fractions.

Pomace and its components were studied by DSC, Figure 2A (top). The dry pomace displayed two weak endothermic peaks at −22 and 26 °C that were associated with the seed fraction since no melting was observed in fibers and skins. The peak at lower temperature can be related to esters of unsaturated fatty acids such as linoleic and oleic acids, while the one at 26 °C was ascribed to the saturated homologues (palmitic and stearic acids). On the other hand, DSC thermorgrams of the Hx fractions recovered from pomace, seeds and skins were well differentiated, Figure 2A (bottom). Hx seed had a sharp peak at −11.8 °C and a broader one around 21 °C, attributed to unsaturated and saturated fatty acids, respectively. On the other hand, Hx skin displayed a single melting at 55 °C. The melting enthalpies associated with the main peaks of each component were 27.4 and 55.0 J/g, respectively. As expected, Hx pomace was a mixture of Hx seed and Hx skin. The relative contribution of each component was calculated from the area of the melting peaks, revealing that ~30% of the Hx pomace came from seeds.

Themogravimetric curves of Hx fractions are displayed in Figure 2B. For Hx seed, the initial weight loss at 229 °C was likely due to the elimination of linoleic acid, while the second one at 301 °C was related to the thermal decomposition of oleic acid [41]. Hx skin displayed a lower initial temperature of decomposition around 230 °C that corresponds to the decarboxylation and/or dehydration/esterification of hydroxylated fatty acids [42]. Finally, the TGA of Hx pomace was very similar to Hx skin, but with a carbonaceous residue close to those of the seeds (~9.5%) that was burned at 487 °C in air. To avoid any thermal degradation during melt polycondensation of Hx fractions, 150 °C was chosen as the temperature to run the reaction. At this temperature, the weight loss was ~1.5% and was related to water removal, either adsorbed or generated by an incipient esterification.

### 3.5. Polymerization of Hx

**Figure 3:**
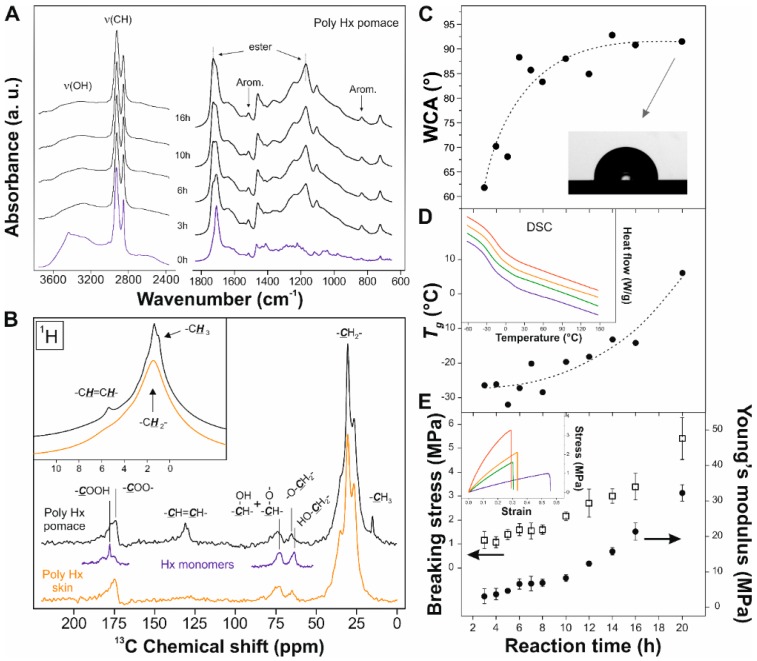
(**A**) ATR-FTIR spectra of polymer films obtained from Hx pomace (poly Hx pomace) at 150 °C in air at the indicated reaction time. (**B**) Solid-state NMR spectra of polymers obtained from Hx pomace (black) and Hx skin (orange). The regions ascribed to –COOH, –CH–OH, –CH–O–, –CH_2_–OH, and –CH_2_–O– of Hx monomers (blue) are also included. (**C**–**E**) Evolution of the water contact angle, glass transition temperature, and tensile parameters, respectively, with the reaction time of the synthesis of poly Hx pomace in air. Inset in **C** shows a typical drop on a surface of poly Hx pomace after 20 h of reaction. DSC (inset of **D**) and stress-strain (inset of **E**) curves correspond to polymers obtained after: (blue) 3 h, (green) 6 h, (orange) 10 h, and (red) 16 h reaction.

The polymerization was chemically characterized by ATR-FTIR spectroscopy, Figure 3A. The esterification was evidenced not only by the development of peaks at 1170 and 1104 cm^−1^ corresponding to ν_a_(C–O–C) and ν_s_(C–O–C) in esters, respectively, but also by the enhancement of the ν(C=O) ester at 1729 cm^−1^ and the reduction of both the broad hydroxyl band (~3300 cm^−1^) and the ν(C=O) acid in the 1710–1700 cm^−1^ range. The infrared spectra showed no relevant modifications above 12 h–14 h, and the reaction was then considered completed. To further investigate the chemical modifications during polymerization, solid-state NMR spectroscopy was carried out, Figure 3B. ^13^C NMR confirmed the formation of the ester by means of the peak at 174 ppm and the one corresponding to primary esters at 65 ppm. Such new signals are accompanied by the disappearance of the peaks at 178 ppm (acid) and 63 ppm (primary alcohol) [42,43,44,45]. The consumption of the acid was verified from the ^1^H spectrum (no peaks above 8 ppm characterizing the free acid). NMR also detected the incorporation of unsaturated monomeric units from the seeds by distinctive double bond (^13^C 131 ppm, ^1^H 5.4 ppm) and methyl (^13^C 15 ppm, ^1^H 1 ppm) signals.

### 3.6. Physical Characterization of Poly Hx

Water contact angle, glass transition temperature (*T*_g_), and tensile parameters were modified with the reaction time, Figure 3C–E, respectively. Their evolution revealed an increase of hydrophobicity, structure rigidity, and toughness as the reaction progressed. Thus, WCA were increased from ~62° for 3 h to ~91° for 20 h of reaction time, while *T_g_* ranged from ca. −26 °C for 3 h to ~8 °C for 20 h of reaction time. Breaking stress and Young’s modulus were also increased from ~1 and ~5 MPa, respectively, for 3 h to ~5.5 and ~32 MPa, respectively, for 20 h of reaction time. These changes can be related to the polycondensation reaction: the consumption of polar groups such as –COOH and –OH can induce higher water contact angles, while the crosslinking by ester bonds can effectively increase the rigidity and toughness of the polymer.

To investigate whether the double bonds from unsaturated fatty acids are susceptible of oxidation, originating new functional groups that can react and act as reinforcing agents of the structure, films of poly Hx pomace and poly Hx skin (with and without unsaturated species from the seeds, respectively) in both inert (N_2_) and oxidative (air) atmospheres were prepared. Mechanical, thermal, and WCA values are listed in Table 3. As observed, the combination of the presence of unsaturated fatty acids from the seeds and the synthesis in air led to the toughest material. In the absence of oxygen, the contribution from the seeds was irrelevant and the mechanical behavior of the polymer was identical to the one obtained from Hx skin. Hence, our hypothesis involves the oxidation of few (C=C) double bonds to –COOH and the formation of additional ester bonds crosslinking the polymer chains and increasing the rigidity. A similar mechanism, in this case involving an oxidative diol cleavage, has been reported in the melt-polycondensation of aleuritic (9,10,16-trihydroxyhexadecanoic) acid [42,46,47]. Spectroscopic data indicate that the oxidation of (C=C) double bonds is not massive, which suggest that it is self-regulated and retarded with respect to esterification because of the diminishment of oxygen diffusion into the bulk, caused by the increment of viscosity as the crosslinking progresses [42].

The series was thermally quite stable, with high maximum decomposition temperatures (T_d max_), as reported for primary polyesters [45,46]. However, the onset of thermal decomposition (T_d 5%_) was slightly lower when unsaturated fatty acids were present in the monomeric mixture. This is related to the oxygen lability of (C=C) double bonds. In the case of poly Hx pomace prepared in air, the decarboxylation of the non-reacted new carboxylic acids formed by oxidation may contribute to the lower decomposition onset temperature. Such polar residual –COOH, preferentially formed at the near surface regions due to the O_2_ presence, may also be responsible for the lower WCA of poly Hx pomace prepared in air.

## 4. Conclusions

Whole tomato pomace (i.e., skin, seeds, and fibers), a cheap, abundant, underutilized, and renewable residue from the tomato fruit processing industry, was characterized and used as a raw material to fabricate bio-based materials. The treatment of the pomace uses common processes and affordable chemicals, such as aqueous solutions of NaOH and HCl, facilitating the scale up to large volume manufacturing. The extraction consisted of an alkaline hydrolysis at moderate temperature followed by neutralization to obtain a mixture of unsaturated and hydroxylated fatty acids with a good yield (~31%, *w*/*w*). The hydrolysis was optimized to reduce costs associated with chemicals and energy consumption. The procedure was compatible with other valorizations of this agricultural by-product, such as the recovery of lycopene and edible proteins. It also left a secondary low-lignin polysaccharide residue (31–32%, *w*/*w*) with potential use in the production of bioalcohols by fermentation.

Cutin-based polymers were prepared from the recovered monomers by melt-polycondensation using no catalyst to preserve innocuousness. Obtained freestanding films were hydrophobic (WCA values close to 90°), infusible (no melting points were detected), amorphous, and thermally stable (decomposition onset temperature above 300 °C). Nevertheless, mechanical properties were improved by the presence of oxygen in the reaction atmosphere, tentatively by the occurrence of moderate crosslinking involving the oxidation of unsaturated fatty acids from the seeds. The control over this secondary reaction, that is, oxygen concentration and temperature, is envisaged as a procedure to tune the physical and chemical properties of polyesters obtained from tomato pomace extracts.

## Figures and Tables

**Table 1:** Fraction and component content of tomato pomace by-product.

Pomace Fraction	Content (%, *w*/*w*)	Components (*)	Calculated Content in Pomace (%, *w*/*w*)
Fiber	15	Polysaccharides (cellulose, hemicelluloses) and lignin (100%)	15
Seed	55	Polysaccharides (cellulose) (19%)	10
		Proteins (29%)	16
		Lipids (unsaturated:saturated ~80:20) (26%)	14
		Others (26%)	14
Skin	30	Cutin (fatty acids, including minor contributions of flavonoids and phenolic compounds) (65%)	20
		Polysaccharides (cellulose, hemicelluloses, pectin) (32%)	9
		Waxes (3%)	1

* Percentages between brackets are from literature.

**Table 2:** Monomeric composition of Hx fractions extracted from tomato pomace and isolated seeds by alkaline hydrolysis.

Monomer			Abundance (Molecular %)	
			Hx Pomace	Hx Seed
Acids	saturated	C_14_ C_16_ C_18_ *p-*coumaric	1.2 4.3 0.9 2.6	0.2 17.1 1.2 -
	**Total**		**9.0**	**18.5**
	unsaturated	9(en)-C_18_ (oleic) 9,12(dien)-C_18_ (linoleic)	11.9 16.1	28.4 52.9
	**Total**		**28.0**	**81.3**
Hydroxy acids	mono-OH	16-C_16_	2.8	-
	di-OH	9,16-C_16_ 10,16-C_16_ 8(9),18-C_18_ 10(11),18-C_18_	2.0 43.3 0.6 1.0	0.2 - - -
	tri-OH	9,10,18-C_18_	1.7	-
	**Total**		**51.4**	**0.2**
Diacids	saturated	C_16_ C_18_	1.8 0.2	- -
	**Total**		**2.0**	**0.0**
Hydroxy diacids	mono-OH	7-C_16_ 8-C_16_	2.1 4.8	- -
	di-OH	6,7-C_18_ 7(9),8-C_18_ 9,10-C_18_	0.5 1.3 0.9	- - -
	**Total**		**9.6**	**0.0**

**Table 3:** Mechanical, thermal and wettability characterization of polymeric films obtained from Hx with and without the contribution from seeds and in oxidative and inert atmospheres.

Polymer	Young’s Modulus (MPa)	Breaking Stress (MPa)	Breaking Strain (%)	Rupture Energy (N mm/mm^3^)	T_d 5%_ (°C)	T_d max_ (°C)	WCA (°)
Poly Hx pomace (air)	21 ± 4	3.4 ± 0.6	33 ± 8	0.5 + 0.1	298	441	91 ± 4
Poly Hx pomace (N_2_)	8.8 ± 0.6	1.3 ± 0.4	19 ± 7	0.15 + 0.08	313	445	102 ± 6
Poly Hx skin (air)	8.9 ± 0.4	1.9 ± 0.2	24 ± 4	0.24 + 0.08	330	448	103 ± 3
Poly Hx skin (N_2_)	9.0 ± 0.6	1.9 ± 0.3	25 ± 3	0.26 + 0.07	335	446	105 ± 3

## Supplementary Materials

The following are available online at http://www.mdpi.com/1996-1944/11/11/2211/s1, Figure S1: IR transmission spectra of Hx fractions obtained by alkaline hydrolysis from (A) tomato pomace and isolated (B) seeds and (C) skins. The 1690–1485 cm^−1^ region is expanded in the inset, Table S1: Infrared bands of tomato pomace and its fractions.
