# Supplementary material for: Valorization of Tomato Processing by-Products: Fatty Acid Extraction and Production of Bio-Based Materials
Source: Materials (Basel). 2018 Nov 7;11(11):2211. doi: 10.3390/ma11112211 (PMC6266337; doi:10.3390/ma11112211)
Supplement: Supplementary file 1 [file materials-11-02211-s001.pdf]

materials

MDPI

Supplementary information

Valorization of Tomato Processing by-Products: Fatty Acid Extraction and Production of Bio-Based Materials

José J. Benítez, Paula M. Castillo, José C. del Río, Manuel León-Camacho, Eva Domínguez, Antonio Heredia, Susana Guzmán-Puyol, Athanassia Athanassiou and José A. Heredia-Guerrero

Table S1. Infrared bands of tomato pomace and its fractions.

Assignment [a]	Tomato Pomace [b]	Skins [b]	Seeds [b]	Fiber [b]	Main Contributor
ν(O-H)/ν(N–H)	3376	3406 (s, br)<br>O–H H–bonded	3369 (s, br)<br>N–H + O–H H–bonded	3400 (s, br)<br>O–H H–bonded	Polysaccharides<br>Adsorbed water<br>Proteins<br>Lignin<br>Cutin
ν(N–H)	3299 (m, sh)	-	3300 (s, br)<br>N–H H–bonded	-	Proteins
ν(–CH=CH–)	3009 (w)	-	3009 (w)	-	Unsaturated fatty acids
$\nu_a(CH_3)$	2952 (w, sh)	-	2952 (w, sh)	2965 (w, sh)<br>Polysaccharides, rhamnose	Fatty acids
$\nu_a(CH_2)$	2927 (vs)	2927 (vs)	2925 (vs)	2925 (m)	Fatty acids<br>Cutin
$\nu_s(CH_3)$	2871 (vw, sh)	-	2871 (vw, sh)	-	Fatty acids
$\nu_s(CH_2)$	2855 (s)	2854 (s)	2855 (s)	2857 (w)	Fatty acids<br>Cutin
ν(C=O) ester	1743 (s)	-	1746 (s)	-	Esterified fatty acids
ν(C=O) ester	1730 (vw, sh)	1733 (s)<br>Cutin	-	1737 (s)<br>bonded ferulic, *p*-coumaric units	Cutin
ν(C=O) ester	1714 (w)	1715 (w, sh)<br>ester H-bonded	-	-	Esterified fatty acids
Amide I	1654 (s)	1656 (vw, sh)	1655 (s)		Proteins
ν(C=C)		1628 (s)	-	-	Phenolics
ν(C=C) + δ(OH)	1630 (w, sh)	-	1630 (w, sh)	-	Fatty acids<br>Adsorbed water
δ(OH)		-	-	1641 (s, br)	Adsorbed water
ν(C–C)	-	1608 (w, sh)	-	-	Phenolics
ν(C=C–C)	-	1586 (w, sh)	-	-	Phenolics
Aromatic skeletal	-	-	-	1597 (w, sh)	Phenolics<br>Lignin

vibration + ν(C=O)					
Amide II	1542 (m)	-	1541 (m)	-	Proteins
ν(C=C–C)		1550 (w)	-	-	Phenolics
ν(C=C–C)	1518 (w)	1516 (m) Aromatics	1516 (w, sh) aromatic ring, G units lignin	1509 (m) aromatic ring, G units lignin	Phenolics Lignin
δ(CH_2) + δ(COH)	1456 (w)	1457 (w, sh) cutin	1457 (m) polysaccharides, fatty acids	1457 (m) polysaccharides	Cutin Polysaccharides Fatty acids
ν(C=C–C)	1445 (w)	1441 (w)	-	-	Phenolics
ν(C-N)			1439 (vw, sh)	-	Proteins
aromatic skeletal vibration + ν(C–N)	1415 (vw)	1416 (vw) Phenolics	1417 (w) phenolics, proteins	-	Phenolics Proteins
aromatic skeletal vibration + δ(CH)	-	-	-	1424 (m)	Lignin
ν(C–N)	1400 (vw)	-	1400 (w)	-	Proteins
δ(CH) + $\delta_s(CH_3)$	1377 (m)	1373 (w)	1379 (w)	1377 (m)	Fatty acids Lignin
δ(OH)	1332 (vw)	1337 (vw)	-	1335 (w)	Phenolics Lignin
δ(CH_2) wagging	1317 (w)	1321 (vw)	1317 (w)	1322 (w)	Cutin Fatty acids
δ(OH)	1262 (w, sh.)	-	-	1268 (w, sh)	Polysaccharides
Amide III + δ(OH)	1239 (s)	-	1237 (m)	-	Proteins Lignin
δ(COH) + ν(C–O) + ν(C–C)		1244 (s, br.)	-	-	Phenolics Cutin
δ(OH)		-	-	1247 (m)	Lignin
ν_a(C–O–C)	1159 (s)	1165 (s)	1161 (m)	1161 (m)	Cutin Polysaccharides
ν_s(C–O–C) ester + asymmetric in-phase ring vibration	1101 (m)	1103 (m)	1116 (w)	1117 (w, sh)	Cutin Polysaccharides
asymmetric ring vibration	-	-	1092 (w)	1104 (w)	Polysaccharides
ν(C–OH) secondary	-	-	1073 (w)	1079 (w)	Polysaccharides
ν(C–O) + ν(C–C)	1065 (s)	1064 (vs)	1055 (s, br)	1052 (vs)	Polysaccharides (hemicellulose)
ν(C–O) + ν(C–C)	1035 (m)	1029 (s)	-	-	Polysaccharides (pectin)
δ(CH) out-of-plane + C–O valence vibration	969 (vw, sh)	-	997 (w, sh)	989 (w, sh)	Polysaccharides (cellulose)
δ(CH) out-of-plane	917 (vw, sh)	922 (vw)	-	-	Phenolics

δ(C_1–H) + anomere COC, CCO, CCH vibrations	899 (vw, sh)	896 (w)	-	896 (w)	Polysaccharides (cellulose)
δ(CH) out-of-plane (γ band)	832 (w)	833 (m)	-	-	Phenolics
ν(C–N)	817 (vw)	-	812 (w)	-	Proteins
δ(CH_2) rocking	722 (vw)	722 (w)	722 (w)	722 (vw)	Cutin Fatty acids
δ(C–OH) out-of-plane	669 (vw)	668 (vw), cellulose	-	669 (w)	Polysaccharides (cellulose)

[a] ν, stretching; δ, bending; a, asymmetric; s, symmetric.

[b] s, strong; m, medium; w, weak; vs, very strong; vw, very weak; br, broad; sh, shoulder.

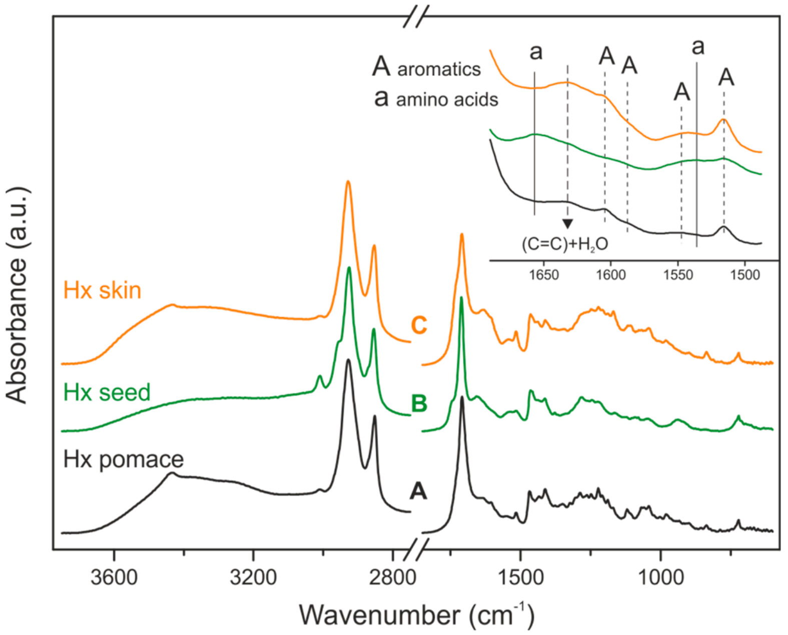

Figure S1. IR transmission spectra of Hx fractions obtained by alkaline hydrolysis from (A) tomato pomace and isolated (B) seeds and (C) skins. The 1690-1485 cm^{-1} region is expanded in the inset.

The calculated Hx^{max} value was higher than the theoretical amount of fatty acids that can be extracted from the skins of the pomace (20%), Table 1. Consequently, other components should be considered to account for the rest (10–12%). Fatty acids from seeds could represent up to a 15% supplement and would justify by themselves such a difference. Actually, the ν(–CH=CH–) peak at 3010 cm^{-1} in the IR spectrum of the Hx pomace is a fingerprint of such contribution, Figure S1A. To quantify the contribution of seeds to Hx pomace, isolated seeds were processed in the same conditions as the pomace, resulting in a dry precipitate after acidification (Hx seed) of about 18% of the initial weight. Considering the abundance of seeds in the pomace (55%), their contribution to the Hx pomace is estimated in about 10%, which accounts for the supplementary recovery. The IR spectrum of Hx seed, Figure S1B, suggests the predominance of aliphatic acids (intense bands of –COOH (1710 cm^{-1}) and –CH_2– (2853 and 2926 cm^{-1}). However, proteins can also be recovered from tomato seeds by alkaline treatment and acidification. In fact, amide I and II bands can be detected in the IR spectrum of Hx seed fraction (upper inset in Figure S1), but their intensity is much more reduced in Hx pomace. The amino acid content in Hx pomace was experimentally determined, according to reported HPLC protocols (Megías, Cortés-Giraldo, Girón-Calle, Vioque, & Alaiz, 2015) to be less than 0.1% (mainly glycine). Hence, Hx pomace is mostly constituted of unsaturated and hydroxyl fatty acids.

Megías, C.; Cortés-Giraldo, I.; Girón-Calle, J.; Vioque, J.; Alaiz, M. Determination of l-canavanine and other free amino acids in Vicia disperma (Fabaceae) seeds by precolumn derivatization using diethyl ethoxymethylenemalonate and reversed-phase high-performance liquid chromatography. *Talanta* **2015**, *131*, 95–98.
